# Chemical Patterns of Proteasome Inhibitors: Lessons Learned from Two Decades of Drug Design

**DOI:** 10.3390/ijms20215326

**Published:** 2019-10-25

**Authors:** Romina A. Guedes, Natália Aniceto, Marina A. P. Andrade, Jorge A. R. Salvador, Rita C. Guedes

**Affiliations:** 1 iMed.Ulisboa and Faculdade de Farmácia, Universidade de Lisboa, 1649-003 Lisbon, Portugal; rominaguedes@ff.ulisboa.pt (R.A.G.); nataliaaniceto@ff.ulisboa.pt (N.A.); 2 Center for Neurosciences and Cell Biology, Universidade de Coimbra, 3004-504 Coimbra, Portugal; salvador@ci.uc.pt; 3 ISCTE – Instituto Universitário de Lisboa/ISTAR-IUL, 1649-026 Lisboa, Portugal; marina.andrade@iscte-iul.pt; 4 Laboratory of Pharmaceutical Chemistry, Faculty of Pharmacy, University of Coimbra, 3000-548 Coimbra, Portugal

**Keywords:** proteasome, proteasome inhibitors, molecular descriptors, fingerprints, chemical space, decision tree, structure-activity relationship

## Abstract

Drug discovery now faces a new challenge, where the availability of experimental data is no longer the limiting step, and instead, making sense of the data has gained a new level of importance, propelled by the extensive incorporation of cheminformatics and bioinformatics methodologies into the drug discovery and development pipeline. These enable, for example, the inference of structure-activity relationships that can be useful in the discovery of new drug candidates. One of the therapeutic applications that could benefit from this type of data mining is proteasome inhibition, given that multiple compounds have been designed and tested for the last 20 years, and this collection of data is yet to be subjected to such type of assessment. This study presents a retrospective overview of two decades of proteasome inhibitors development (680 compounds), in order to gather what could be learned from them and apply this knowledge to any future drug discovery on this subject. Our analysis focused on how different chemical descriptors coupled with statistical tools can be used to extract interesting patterns of activity. Multiple instances of the structure-activity relationship were observed in this dataset, either for isolated molecular descriptors (e.g., molecular refractivity and topological polar surface area) as well as scaffold similarity or chemical space overlap. Building a decision tree allowed the identification of two meaningful decision rules that describe the chemical parameters associated with high activity. Additionally, a characterization of the prevalence of key functional groups gives insight into global patterns followed in drug discovery projects, and highlights some systematically underexplored parts of the chemical space. The various chemical patterns identified provided useful insight that can be applied in future drug discovery projects, and give an overview of what has been done so far.

## 1. Introduction

Cancer is a complex, aggressive, and heterogeneous disease that affects a large proportion of the population throughout the world, yet treatment success is still challenging and modest. Recent data estimate 18.1 million new cases and 9.6 million deaths due to cancer in 2018 [1]. 

The ubiquitin-proteasome pathway is responsible for 80% to 90% of eukaryotic intracellular protein degradation, controlling crucial regulatory proteins associated with cell growth, differentiation and apoptosis in cancer cells [2,3,4,5].

Over the past 15 years, proteasome inhibitors (PIs), namely bortezomib, carfilzomib and ixazomib, have significantly improved the overall survival and quality-of-life for multiple myeloma (MM) patients, representing the backbone of the treatment of this cancer [6]. However, a significant percentage of MM patients do not respond to PI therapies; most patients exhibit resistance (innate or acquired) leading to disease relapse and, consequently, to an ever growing need for new alternative therapeutic options for targeting cancer [7,8,9,10].

Two decades of proteasome inhibitors development efforts generated a wealth of unexplored information on proteasome inhibition and an exhaustive analysis of the publicly-available chemical and bioactivity data is yet to be carried out. Detailed knowledge of what drives activity in proteasome inhibitors is the key to accelerate the understanding of chemical and biological information vital to design more efficient and selective drugs.

**Figure 1:**
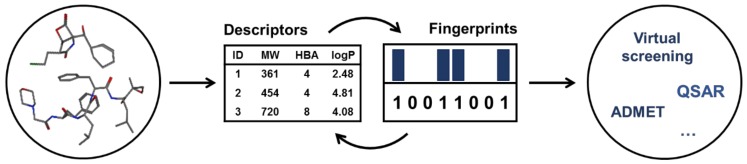
Molecular descriptors and fingerprints are examples of strategies that allow researchers to extract important information about compounds that can be used in additional computer-aided drug design techniques, such as virtual screening, quantitative-structure-activity relationship (QSAR) and prediction of absorption, distribution, metabolism and excretion-toxicity (ADMET) [15].

Different studies have been published in the last two decades, trying to establish structure-activity relationships (SARs) but these are performed on few and/or low-diversity sets of compounds (Chiba, Matsuda & Ichikawa [11]; Hovhannisyan et al. [12]; Macherla et al. [13]; Zhu et al. [14]) and such studies are largely empirical medicinal chemistry analyses. However, a multitude of different ways to define compounds exists, such as drug-likeness, molecular descriptors and structural fingerprints (e.g., MACCS, ECFP), that can capture molecules under different perspectives (Figure 1). These have been widely used to characterize the already known active compounds and correlate chemical patterns with experimental data, effectively uncovering structural/physicochemical determinants for activity and specificity across multiple therapeutic applications. This allows deriving knowledge which can be used in the form of general rules to filter compound databases with billions of compounds and exclude less promising candidates.

The aim of this work is to perform a comprehensive analysis of a full dataset comprising 680 small-molecule proteasome inhibitors, developed in the last two decades to generate new knowledge priceless for new drug discovery campaigns.

### 1.1. The Proteasome: a “Millennial” Target

The importance of the proteasome in cancer is unquestionable. The ubiquitin-proteasome system (UPS) plays a fundamental role in adenosine triphosphate (ATP)-dependent protein degradation in the cytoplasm and nucleus of eukaryotic cells, regulating a wide variety of cellular pathways, namely cell cycle control, apoptosis, DNA repair, transcription, immune response and signaling processes via the degradation of cellular key players (e.g., cyclins or tumor suppressors like p53) [4,16,17]. 

**Figure 2:**
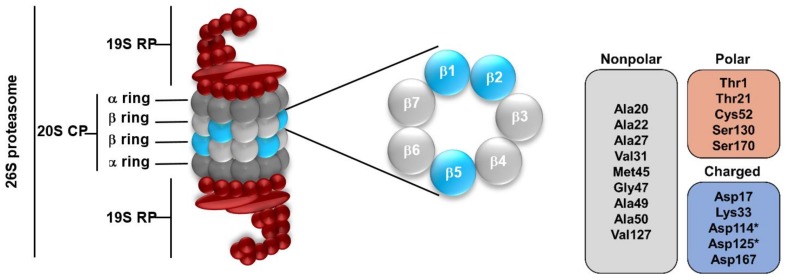
(Left) The eukaryotic 26S proteasome and the three catalytic subunits of the β ring: β1, β2 and β5. (Right) Residues that line the chymotrypsin-like (CT-L) catalytic site, on which we focus in this paper’s analyses. Residues with * belong to the β6 subunit, otherwise they belong to the β5 subunit.

The key component of the UPS is the 26S proteasome (Figure 2), particularly the 20S core particle (also designated as “20S proteasome” or simply “proteasome”), which is composed by four heptameric rings (two α rings and two β rings organized as α-β-β-α) [18,19,20,21,22,23]. In the eukaryotic proteasome, only three out of the seven different β subunits (β1, β2 and β5) have proteolytic activity because they bear N-terminal proteolytic active centers due to the existent Thr1 N-terminal residues (being these protected by propeptides before the core particle maturation), in which the hydroxyl group (Thr1Oγ) acts as a nucleophile in the hydrolysis of the peptide bond [2,24,25,26,27,28,29]. Moreover, each of the three proteolytic β subunits has differences when binding to substrates and activity performed: The β1 subunit presents “caspase-like” (C-L) activity, and cleaves peptide bonds after acidic amino acids; β2 subunit has “trypsin-like” (T-L) activity, and cleaves peptide bonds after basic amino acids; β5 subunit has “chymotrypsin-like” (CT-L) activity, and acts after neutral amino acids [20,21,22]. 

### 1.2. Proteasome Inhibitors

**Figure 3:**
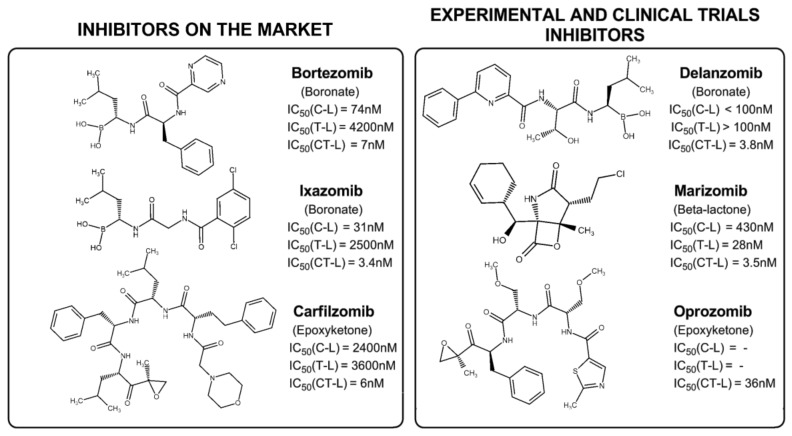
Clinical or pre-clinical Human 20S proteasome inhibitors. Note that, even though largely considered an experimental compound, marizomib has received approval as an orphan drug for glioblastoma. Additional details can be found in Table 1. C-L: Caspase-like active site; T-L: Trypsin-like active site; CT-L: Chymotrypsin-like active site. References for IC_50_ values: Bortezomib and carfilzomib, Demo et al. [33]; ixazomib, Kupperman et al. [34]; carfilzomib, Demo et al. [33]; delanzomib, Piva et al. [35]; marizomib, Chauhan et al. [36]; oprozomib, Zhou et al. [37].

In vitro studies have shown that malignant cells exhibit greater susceptibility to proteasome inhibition compared to normal cells, due to their overexpression of proteasome activity (Dou & Li) [30]. Fortunately, the extensive research in the field of proteasome inhibitors during the last two decades leads already to three drugs on the market mainly used in the treatment of MM-bortezomib (Velcade^®^), carfilzomib (Kyprolis^®^) and ixazomib (Ninlaro^®^) (Figure 3). Bortezomib is also used in the treatment of mantle cell lymphoma (MCL) [31,32].

Proteasome inhibitors have improved significantly the survival and quality of life of patients with MM [38]. Also, marizomib has received “orphan drug” designation to the use in the treatment of gliobastoma in the USA, and also for the treatment of MM in the USA and the European Union (EU) [39,40]. Table 1 gives an overview of some of the most important proteasome inhibitors, their structural classes, administration route, market authorizations and the phase of ongoing clinical trials (and respective diseases).

All clinically-available proteasome inhibitors target at least the CT-L catalytic subunit. So, this study will only focus upon the inhibition of this catalytic subunit. The most potent proteasome inhibitors act in the nanomolar range (IC_50_ values for bortezomib, carfilzomib, ixazomib and marizomib are, respectively, 7 nM [33], 6 nM [33], 3.4 nM [34] and 3.5 nM [36]). The functional and biological significance of the different co-inhibitory patterns of the available proteasome inhibitors are still unknown.

Since this is a very active field of research, many other proteasome inhibitors are already in clinical trials, like oprozomib (an epoxyketone) and delanzomib (a boronate) (Table 1). However, despite some advances from academia and the pharmaceutical industry, more than fifteen years have passed since United States Food and Drug Administration (FDA) approval of the first proteasome inhibitor to treat MM and MCL. This is evidence of how challenging it is to find new therapeutic options for the modulation of proteasome activity, which creates an opportunity to use computational methods which can provide invaluable insight.

In this study, we focus on a data-driven strategy, whereby we have identified the important structural and physicochemical determinants that can guide the future discovery of new lead compounds and tune their pharmacological properties, saving time and experimental effort. 

We shortly introduce and apply some fundamental concepts, e.g., molecular descriptor dimensionality, fingerprint description and activity landscapes that will allow us to find some correlations with activity. A step-by-step analysis of the proteasome inhibitors discovered (approved, in clinical trials or in development) during the last two decades will be presented and comprehensively analyzed.

## 2. Material and Methods

### 2.1. Data Pre-Processing

This study focuses on the analysis of several properties and descriptors of a pool of inhibitors of the human 20S proteasome that act in the CT-L active site. A dataset comprising 680 compounds with inhibitory activity in the β5 catalytic subunit (SI1) was assembled and curated using data retrieved from available literature (2000–February 2019), patents and from ChEMBL24.1 [41]. In this study we exclusively collected half maximal inhibitory concentration (IC_50_) data originated from biochemical assays, therefore excluding any sort of indirect measurements originated from phenotypic assays. The corresponding SMILES retrieved from ChEMBL were converted to 3D structures with Molecular Operating Environment (MOE) [42] software (Corporate Headquarters, 910-1010 Sherbrooke St. W., Montreal, QC H3A 2R7 Canada).For all of the other compounds found in the literature, structures were drawn in MOE2018.08.02. All the compounds included in this study were previously protonated with the Protonate-3D tool (pH = 7.4 (slightly alkali), 310.15 K) and partial charges were assigned using the MMFF94x force field as implemented in the MOE software package. Compounds were further energy-minimized (via the “Energy Minimize” tool of MOE 2018.08.02). The dataset was curated by a visual inspection of each chemical structure, removing duplicates and taking a special care with tautomers and isomers. The dataset was divided into four classes of proteasome inhibitory activity (IC_50_ values between 0.08 and 150,000 nM), namely High activity: Class A, 283 compounds (0.08 ≤ IC_50_ ≤ 50 nM); Moderate activity: Class B, 126 compounds (50 < IC_50_ ≤ 500 nM); Low activity: Class C, 208 compounds (500 < IC_50_ ≤ 10,000 nM); and Very Low activity: Class D, 63 compounds (10,000 < IC_50_ ≤ 150,000 nM).. Class D compounds are included in this study as a negative control class as they show what could largely be considered marginal activity. This class is intended to represent the chemical space of “unspecific activity” in which the proteasome might not even be the main target. In practical terms, this is the space that typically one would want to avoid during drug design.

### 2.2. Computation of Molecular Descriptors

Molecular descriptors were calculated using MOE 2018.08.02 (“QuaSAR-Descriptor”), which included several traditional molecular descriptors, such as structural keys, physicochemical properties, 3D molecular features and also some pharmacophore-based descriptors [43]. The selection of calculated molecular descriptors was based upon drug-likeness/lead-likeness rules, desirable drug features, namely water solubility and descriptors directly related with medicinal chemistry properties [44,45,46]. A total of 21 descriptors were ultimately obtained and used to carry out all analysis in this work in order to maximize the interpretability of results: The number of atoms (a_count), number of hydrophobic atoms (a_hyd), number of hydrogen bond acceptor atoms (HBA), number of hydrogen bond donor atoms (HBD), number of aromatic bonds (b_aro), number of single bonds (b_single), number of double bonds (b_double), number of triple bonds (b_triple), number of rotatable bonds (b_RotN), number of rotatable single bonds (b_1RotN), number of rings (rings), number of chiral centers (chiral), molecular weight (MW), logarithm of the octanol/water partition coefficient (logP(o/w)), water solubility (logS), topological polar surface area (TPSA), molecular refractivity (MR), Lipinski druglike test (lip_druglike), number of violations of Lipinski’s Rule of Five (lip_violation), presence of potential toxic groups (mutagenic) and presence of reactive groups (reactive).

### 2.3. Chemical Space, Similarity and Scaffolds Analysis

The distribution of classes within the chemical space defined by the 21 descriptors annotating the dataset was visualized using t-distributed Stochastic Neighbor Embedding (t-SNE) [47]. This is one of the most successful multi-dimensional scaling tools in conserving relative distances when compressing high-dimensional space into low-dimensional space [47]. The 21-dimension matrix was compressed into two dimensions, using the default parameters set for t-SNE implementation in Scikit-learn [48]. The different classes were also characterized in terms of scaffolds, which were obtained using the Murcko Scaffolds [49] decomposition available in RDKit [50]. The similarity between scaffolds was measured using the Tanimoto coefficient (Tc) [51] computed over Morgan fingerprints, the Extended-Connectivity Fingerprints [52] implementation in RDKit (calculated for a radius of 2 atoms, folded over 1,024 bits). RDKit was used to perform similarity calculations, calculate Morgan fingerprints and Murcko Scaffolds.

### 2.4. Statistical Analysis and Machine Learning

Statistical analysis of molecular descriptors was performed with IBM SPSS Statistics [53] version 25 and with SciPy [54]. The statistical data visualization was performed with Seaborn [55] and Matplotlib [56]. The data analysis workflow was assembled in Jupyter [57]. In order to derive useful rules that determine activity, a decision tree was trained, using Scikit-learn implementation in Python, to classify the activity data using the 21 physicochemical descriptors under study in this work. In order to allow for sufficiently general patterns to arise, tree depth was limited to seven, and the minimum number of samples per leaf was set to five. The most relevant branches were then extracted for detailed analysis.

## 3. Results and Discussion

During the drug design process, to assess the potential druggability of a compound as a new starting point, some initial rules like drug-likeness (as defined by Lipinski and colleagues in 1997 [58]), lead-likeness (as defined by Oprea [59,60]) and known drug space, are usually applied. In general, the rules to characterise/filter compound collections are based upon physicochemical parameters, e.g., logP, logS, MW, TPSA, MR, RotN, HBA and HBD, among others [61]. As a result, it would be interesting to assess how proteasome inhibitors are positioned with respect to the typically expected “drug-like” chemical space.

To do so, we performed a comprehensive analysis of the 680 small-molecules proteasome inhibitors dataset spanning a large range of activity (0.08 nM < IC_50_ ≤ 150,000 nM). This entailed looking at chemical space distribution, scaffold similarity and the extraction of chemical rules with machine learning, using either direct structural information (scaffolds and Morgan fingerprints) or being based on 21 general molecular (QuaSAR) descriptors. This set of analyses was ultimately employed to produce meaningful chemical patterns that are correlated with proteasome inhibitory activity. The dataset comprises only human proteasome activity data for the CT-L catalytic site, as this is the only site with the largest number of compounds, and is divided into four classes. Full data are available in Table S1.

Analysis of chemical space distribution and the scaffold similarity of proteasome inhibitors. First, we analyzed human proteasome inhibitors’ chemical space distribution. To address this analysis, chemical space was defined by the t-SNE calculation applied to the 21 calculated molecular descriptors listed in the Supplementary Information (Table S1). The proximity in a t-SNE plot is relative in nature, and the distance between points simply represents relative proximity (i.e., it is not a direct scaling from real distances in the 21 dimensional space).

**Figure 4:**
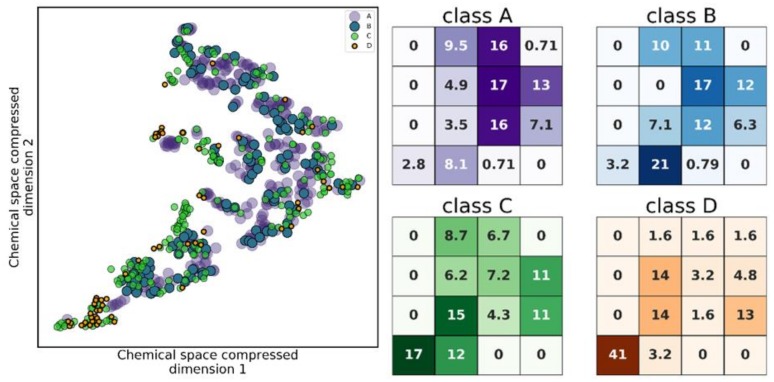
(Left) Chemical space of the four half maximal inhibitory concentration (IC_50_) value classes, where an uneven distribution of classes can be seen. (Right) Density of data points per class, represented as % points from a given class found in a specific region of the plot, with respect to the total amount of points from that class. Class A preferentially populates the top-right quadrant of the plot, while C and D populate the lower-left corner.

In Figure 4 (left) the difference of distribution of the four activity classes is easily perceptible, with the most active compounds (class A, purple) having close to half of its compounds (~47%) in the top-right quadrant of the plot, while the least active compounds (class D, orange) are mainly concentrated in the bottom-left quadrant (~58% of class D compounds), as quantified in Figure 4 (right). Classes B (blue) and C (green) have a more disperse distribution, but show some higher density areas close to the bottom-left corner. This indicates that class D, for example, is fundamentally different to the remaining classes from a structural and physicochemical level, and therefore supports the existence of a SAR in the proteasome inhibitors developed in the last 20 years. Additionally, no particular cluster was formed with a single class, which means there is no particular location in chemical space (at least one that is defined by the descriptors that we considered) which is reserved to one single class. This observation indicates that carrying out compound selection biased for physicochemical similarity to known inhibitors might be a good initial filter to enrich the set of compound candidates with active hits.

**Figure 5:**
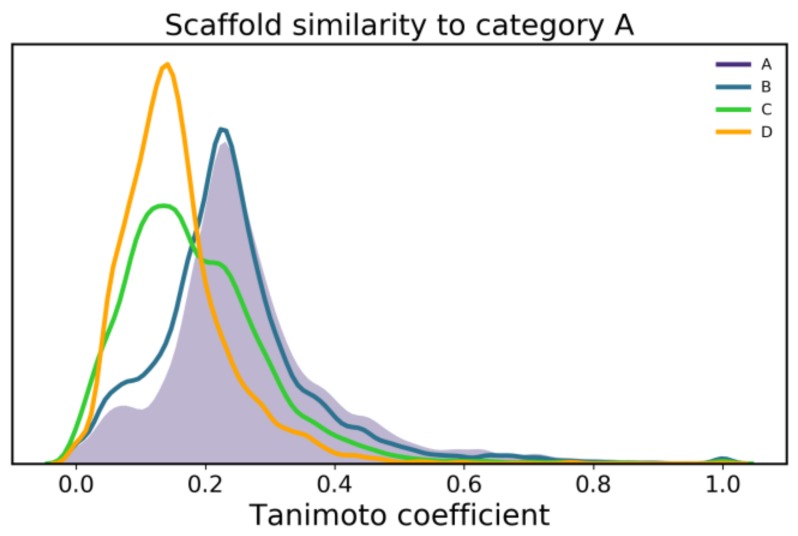
Scaffold similarity between the four IC_50_ classes of proteasome inhibitors.

Next, we wanted to investigate what characteristics are responsible for the differences between classes in the chemical space distribution. Upon applying the Tc to characterize the similarity between Murcko scaffolds within each class, we observed a high diversity in the dataset, not just within each class, but also between the different classes, with most pairs of compounds showing a Tc below 0.5 that represents a considerable dissimilarity (Figure 5). Nonetheless a clear trend of correlation between activity and similarity is observed.

The scaffolds of class B differ negligibly from class A, however, class C shows a two-peak distribution which suggests it contains scaffolds similar to both classes A/B, as well as with lower activity compounds (class D). This is probably a result of the practical strategies applied in medicinal chemistry where inactive structures are modified/derivatized to meet or better resemble substructures seen among actives, thus creating this transition from A to D. As concluded from the chemical space observation, this also shows that functionalizing scaffolds from more potent compounds (class A/B) might be a feasible initial strategy to find new actives. However, selected scaffolds should also be sufficiently dissimilar to the lower activity scaffolds, effectively avoiding the right-end tail of the class D (i.e., the most similar scaffolds to A, which are still largely inactive).

**Figure 6:**
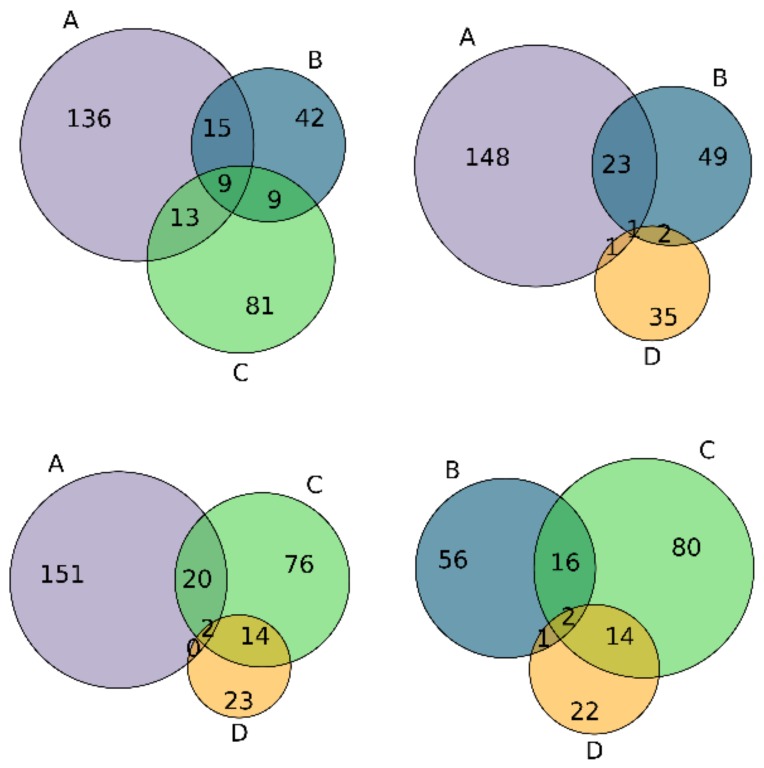
Venn diagrams representing the scaffolds common to the four IC_50_ classes.

Beyond looking at similarity, we wanted to address scaffolds that are actually shared between classes, as these are scaffolds which are more prone to an unexpected loss of activity from functionalization. The Venn diagram of scaffolds between different classes (Figure 6) confirms the scaffold differences between the four classes, where there is a significant decrease in the number of common scaffolds when comparing the overlap of class A vs. B (24 scaffolds), A vs. C (22 scaffolds) and A vs. D (2 scaffolds). Furthermore, the four classes have only one scaffold in common. Special attention should be paid to the 22 shared scaffolds (lower-left plot, Figure 6) shared between A and C/D, which are potentially more prone to drastic changes in activity, and which can be used to prevent loss of activity or search for gain in activity. In Table S2, the comprehensive list of common scaffolds between the several classes is provided.

**Figure 7:**
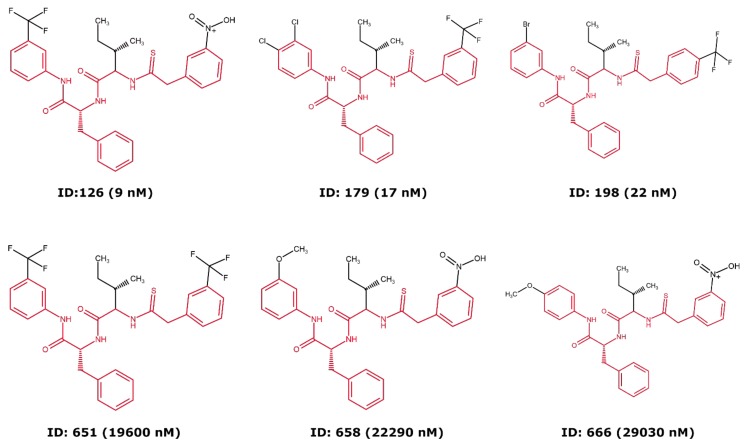
Compounds with the main, largest scaffold shared between class A (top) and D (bottom).

Figure 7 shows the only sufficiently large scaffolds shared between classes A and D. Inspecting cases such as this one can give insight into what chemical transformations drive activity, and the analysis of this particular scaffold reveals that small changes in the position, volume and/or polarity of the substituents at extreme ends of the scaffold induces a dramatic shift in activity. For example, a single change from a trifluoromethhyl (compound 126, Figure 7) to a methyl ether (compound 658) in the *meta*-position of one of the benzene rings drives a 2500-fold loss in activity.

### 3.1. Building a Decision Tree to Derive Chemical Rules That Determine Proteasome Inhibition

**Figure 8:**
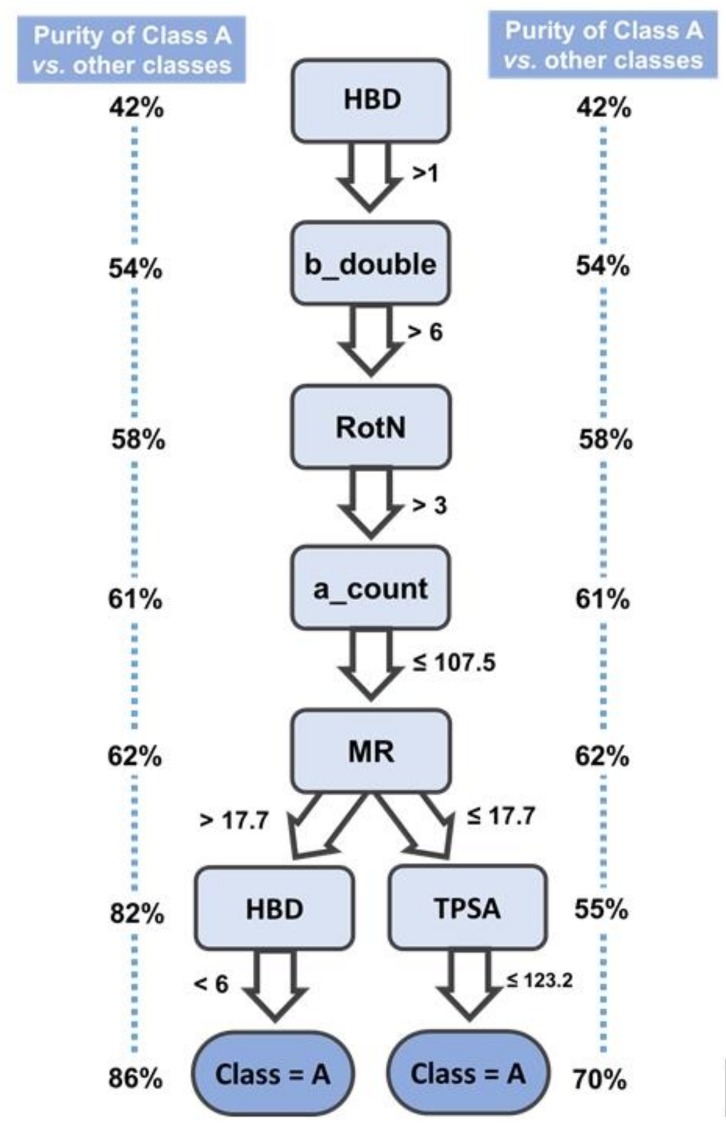
Schematic representation of the most important decision rules for the classification of class A compounds within the decision tree built from the proteasome inhibitors dataset.

In order to rationalize the difference between activity classes, a decision tree (Figure S1) was built using the full dataset and the calculated descriptors. This is a strategy to exhaustively find the most meaningful chemical patterns that determine proteasome inhibitory activity. Here, a decision tree algorithm was tasked to find the decision splits that are able to separate, with the highest efficiency, the four inhibitory classes. Generally speaking, at the top of the three the algorithm looks through all available descriptors and picks the one that allows for the best numerical threshold for separation between classes, which produces two child nodes. Next, the algorithm looks again for the best descriptors to split each of the child nodes into two new nodes each. The process is iterated until maximal separation is found (or until stopping parameters, like minimum compounds per node, are reached) [62]. The obtained decision tree revealed two decision rules which are responsible for the classification of a large fraction of the most potent activity class A (63.6% of class A compounds). These are defined in the decision tree scheme in Figure 8, and additional information on class ratios in the different nodes of the decision rules is provided in Figure S1.

Both rules efficiently separate class A from the remaining three classes, which is shown by the high purity level (i.e., compounds of class A/All compounds) achieved after the last decision split in each rule (e.g., 86% and 70%, respectively). These can be therefore used as general rules of thumb when searching for high potency compounds. Generally speaking, these rules indicate that in order for a compound to be a potent proteasome inhibitor, it must have some sort of hydrogen bonding ability; a considerable amount of double bonds must exist, but be accompanied by sufficient flexibility. A surprisingly high maximum atom count (107.5) requirement was also found in these rules, however this might just be a result of the general “molecular obesity” trend in the last decades [63]. Compounds must also show some polarity, seen by the fact that a lower limit is set for MR; however this last feature is not very informative, seeing as many drugs span between 40 and 130 m^3^·mol^−1^ in MR [64,65]. Finally in order to be active, a compound must obey to the maximum allowance for HBD atoms of five, or show a limited TPSA. Due to the high coverage combined with the high degree of separation between class A and the other three classes, this rule can be useful to bias compound selection prior to virtual screening, for example.

### 3.2. Analysis of Key Molecular Descriptors and How They Relate to Proteasome Inhibition

The decision tree generated (Figure 8) uncovered some of the key molecular descriptors that set apart the most active compounds from medium-to-lower activity compounds. However, it might be useful to examine how these and related descriptors behave across all classes (no longer focusing on one class vs. rest). As a result, we will now provide an overview of the molecular descriptors calculated for all of the classes in the entire dataset, where we characterized each one with respect to their distribution and spread in each activity class. The performance (mean and medium values) of each descriptor was evaluated for each of the classes individually, and whenever possible correlations were established between the variation of the descriptor value with the class of proteasome inhibitory activity. Emphasis was placed on the key descriptors in the decision tree. A table with descriptive statistics can be seen in Table S3.

### 3.3. Size Descriptors (MW, Number of Atoms)

**Figure 9:**
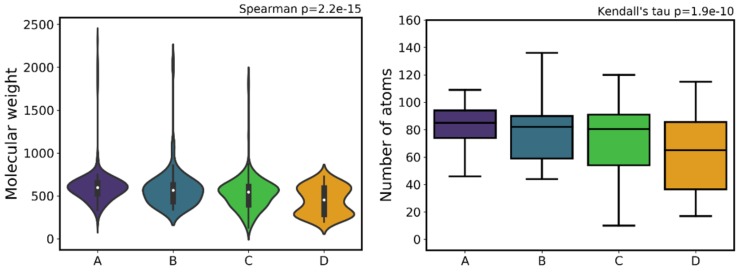
Distribution of Molecular Weight (MW) (left) and number of atoms (a_count) (right) across the different activity classes.

One of the most important descriptors whose profile has been changing in the recent years of drug development is MW, which is due, for example, to its influence in pharmacokinetics and off-target effects [43,66]. However, in this particular case, it has become difficult to infer with confidence a correlation between this descriptor and compounds activity, mainly due to a wide distribution MW range, from 129 g∙mol^−1^ (compound 616, class C) to 2315 g∙mol^−1^ (compound 190, class A). The distribution plots in Figure 9 (descriptive statistics in Table S3) show that the mean and medium values of MW tend to decrease from class A do class D (607 to 443 and 596 to 452, respectively).

Interestingly, and directly related to MW, one of the descriptors that proved key in the decision tree was the number of atoms (a_count). In a general way, a higher number of atoms is correlated with a higher MW, but of course the type of atoms present in the molecule will determine differences in the MW values. In this case, we see a significant decrease of the mean and median number of atoms from class A to class D (86 to 60 and 85 to 65, respectively).

### 3.4. Hydrogen-bonding Descriptors (HBA, HBD)

As expected, due to the importance of hydrogen bonds, particularly HBD, these were found to be crucial to identify very active compounds within the decision tree. Hydrogen bonds influence the molecule reactivity, modifying the electron distribution of the environment of the electron-donor atom, influencing biological reactions, namely membrane permeability and drug action, through the stabilization of the ligands [43,67,68]. This type of interaction is particularly important in the case of proteasome inhibition, since, besides the van der Waals interactions (which are predominant in number), hydrogen bonds are essential for the stabilization of the ligand in the CT-L active site, mainly due to the existence of polar neutral amino acids (e.g., Thr and Tyr) and nonpolar residues (e.g., Ala, Gly) among the residues that line the catalytic site (Figure 2). For example, bortezomib, carfilzomib and ixazomib establish HBA interactions with Thr21 and Ala49, performing also HBD interactions with Thr21 and Gly47, among other residues [69].

**Figure 10:**
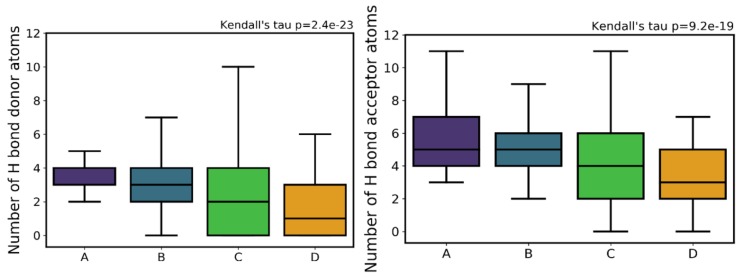
Distribution of hydrogen bond donor (HBD) atoms (left) and hydrogen bond acceptor (HBA) atoms (right) across the different activity classes.

Both HBA and HBD show an overall decrease that follows the decrease in activity (i.e., going from A to D) in all distribution measures (Table S3), particularly evident by the decreasing median lines of the boxplots in Figure 10. Both mean and median values decrease from class A to class D (5.72 to 3.4 and 5 to 3, respectively, in HBA; 3.9 to 1.6 and 4 to 1, respectively, in HBD), indicating an overall trend to lose activity with a lower HBD count. This was an expected pattern given that the loss in the ability to establish H-bonds limits the compound’s ability to interact with and be stabilized in the proteasome active site, and therefore this loss of activity as a function of HDB count is not surprising. 

Comparing the boxplot of class A for HBA versus HBD (Figure 10), HBA has clearly a much larger dispersion of the values (50% above the median) than HBD (which has a higher concentration of values less than or equal to the median).

The fact that even lower activity compounds show some hydrogen-bonding ability is likely a result of the clear bias towards selecting peptidomimetic compounds for screening. In addition, vinyl sulfones were introduced by Bogyo et al. in 1997 [70] as a scaffold of irreversible inhibition, where it was suggested that the double bond of the vinyl sulfone moiety reacts with the hydroxyl on the threonine in the active site. Later in 2008, Baldisserotto et al. [71] in addition showed that compounds bearing α,β-dehydro-phenylalanine are good substrates for the catalytic threonine. Both scaffolds contain a significant amount of HBA and HBD atoms and, as they have likely influenced future compound selection, this explains the observed distribution of HBA/HBD counts. An example of such influence is the study by Akhlaghi, Daeihamed, & Abdolmajid [72] in 2018, where the authors explicitly built upon one of these scaffolds.

### 3.5. Shape and Surface Descriptors (MR, TPSA)

**Figure 11:**
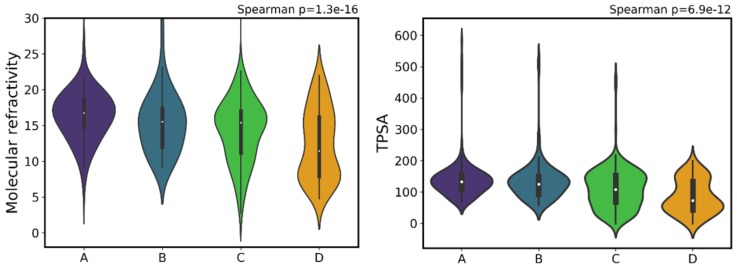
Distribution of molecular refractivity (MR) (left) and topological polar surface area (TPSA) (right) across the different activity classes.

Two descriptors present in the relevant decision paths within the decision tree encode information on shape and surface properties: MR and TPSA. MR encodes information about molecular volume and polarizability, being used as a measure of the binding force between polar portions of an enzyme and its substrate [43]. MR can be defined as the volume of the substance taken up by each mole of that substance, being calculated through the Lorenz–Lorentz equation [73,74] (also known as the Clausius–Mosotti equation) and expressed as m^3^∙mol^−1^ [43]. Bahmani et al. [75] define the polar surface area (PSA) of a molecule as “the sum of the contributions to the molecular surface area of polar atoms such as oxygen, nitrogen and their attached hydrogens”. However, a faster and larger equivalent method to calculate this descriptor is the TPSA. This topological descriptor combines shape and electronic information. Both TPSA and MR slightly increase as bioactivity increases (Figure 11). This is particularly evident by comparing the white center-point (median) of class C with the remaining classes. According to the values specified by the Veber rules [76] and the Rule of Three by Congreve et al. [77], the drug-like benchmark value for the PSA should be ≤ 140 Å^2^, and the lead-like reference value should be ≤ 60 Å^2^, which coincides with the lower and upper ranges for class A. The PSA (similar to TPSA) value defined as an upper limit of the drug-like space is 140 Å^2^, because it is the highest value at which oral absorption occurs (at higher values, the compounds tend to be poor at permeating cell membranes). In the case of compounds that should penetrate the blood-brain barrier, the PSA must be lower than 90 Å^2^ [75,76,78,79]. This is not important in the case of MM, but could be very important in other tumors.

As seen for other descriptors, these recommended filters probably influenced the compounds selected for experimental testing, which explains that all classes show similar upper limits to their corresponding TPSA distribution Figure 11. Regarding MR, the increase in volume and polarizability can be explained by the fact that the typical inhibitor structure has been proposed to require an electrophilic trap from as early as 2000 [80], which increases functionalization with more bulky polar groups such as sulfones, boronates and halogens [81,82], and consequently increases polarizability and volume. Nonetheless, there are outliers in class A with remarkably high TPSA, more than four times larger than the 140 Å^2^, which means that, when it comes to activity alone, much larger polar surface area is allowed while still maintaining excellent inhibitory capability. The same scenario is seen for MR, where outliers in class A show that a large volume is allowed.

### 3.6. Compound Flexibility (Double Bonds, Rotatable Bonds)

**Figure 12:**
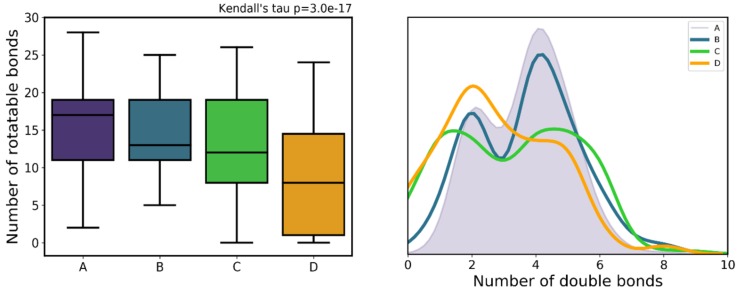
Distribution number of rotatable bonds (left) and number of double bonds (right) across the different activity classes.

**Figure 13:**
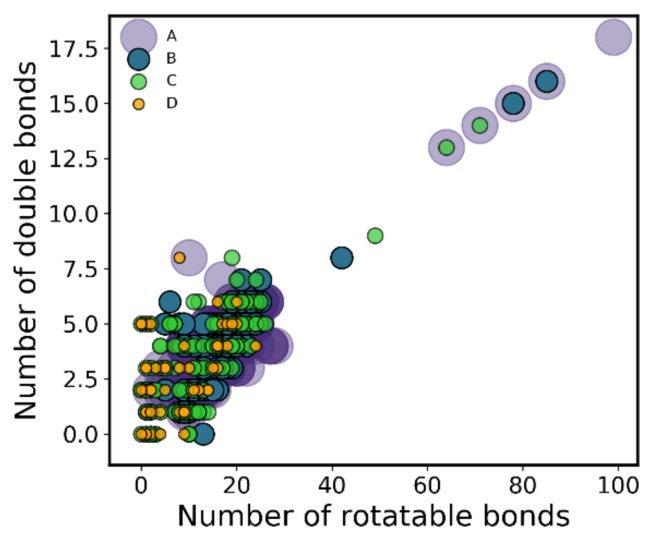
Correlation between double bond count and rotatable bond count for the full dataset. Each point corresponds to a compound, as it is colored by the compound’s class membership.

As uncovered by the decision tree, the number of double bonds and rotatable bonds are relevant predictors of high versus low proteasome inhibitory activity. Further inspection of the distribution of these descriptors across activity classes revealed a SAR across classes, beyond just the separation of class A versus remaining classes, with both variables increasing as a function of activity (Figure 12). Here, the activity of proteasome inhibitors appears to follow a balanced flexibility-rigidity, where, as both rotatable bonds and double bonds increase, activity increases as well. It appears that an increasing amount of rotatable bonds is effectively being offset by an also increasing number of double bonds (Figure 13), which can mean that, in order to bind to the active site, flexibility must exist, but only to some extent. The presence of multiple double bonds in compounds highly influences molecular properties, rigidity and reactivity [43]. Another reason for this correlation between double bonds and rotatable bonds might simply be a result of multiple classical inhibitors showing somewhat of an elongated structure with multiple peptide bonds. This, alongside the knowledge that inhibitors are purposefully designed to act as peptidomimetics in order to mimic natural peptide substrates, might have biased medicinal chemists to add terminal groups to previous less active compounds through peptide (or peptide-like) bonds. This effectively would have increased both the number rotatable bonds, while also adding the double bond within the peptidic bond. In fact, double bonds can be mainly found in the carbonyl group of the peptide chains of some molecules. However, it is worth pointing out that, for example, while marizomib has almost as many double bonds as it does rotatable bonds, carfilzomib shows only a small number of double bonds with respect to the number of rotatable bonds. As they show essentially similar bioactivities (3.5 and 6 nM), it shows the observed flexibility–rigidity balance obviously does not single-handedly determine activity.

### 3.7. Physicochemical Properties (LogS, LogP)

Both solubility and lipophilicity are generally important properties to control during drug development, and are usually measured as logS and logP. However, for the data gathered in the last 20 years on proteasome inhibitors, no statistically significant trend was observed between activity and either logS or logP. The same pattern was found by Shultz [66] from the analysis of approved oral drugs from 1998 to 2017. This indicates that, regardless of activity, all compounds were kept within the same range of values, which corresponds to relatively high lipophilicity, on average. This is in line with the fact that ever since the 1990s, it became well established that both low and high lipophilicity might be associated with poor ADMET properties [83].

**Figure 14:**
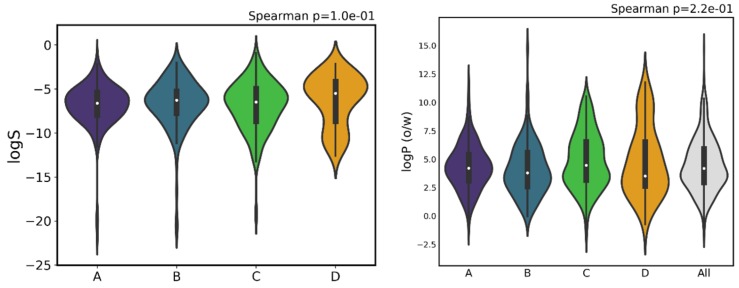
Distribution of logS (left) and logP(o/w) (right) across the different activity classes.

Rules such as Lipinski’s Ro5 established an upper boundary for logP (set at 5) to avoid absorption issues solubility/absorption and in vivo toxicity [61,78,84,85]. However, studies such as Lipinski’s 2000 paper [86] also called attention to potential problems in having too low a logP, as also did a seminal work in 2009, where Keserü and Makara [87] recommend the use of lipophilic ligand efficiency (LLE), which essentially corrects for activity simply caused by increased size and lipophilicity. From this point on, a range for lead-like compounds of −3 < logP < 3 has been proposed which certainly influenced posterior drug development. It is worth pointing out that the fact that logP maintains roughly the same distribution in Figure 14, regardless of activity class, might be an indication of compliance to parameters such as LLE, since is it otherwise very common to observe trends of direct correlation between activity and lipophilicity.

### 3.8. Analysis of Drug-likeness of Two Decades of Proteasome Inhibitors

The search for new hits to enter drug discovery pipelines often starts with a hit-finding campaign in which large collections of available compounds are screened. This step is critical to drug discovery success to assure the tractability of these collections and decrease the attrition rate of drug development candidates in reaching the market. Some initial rules like drug-likeness, lead-likeness and known drug space coverage are often applied to facilitate the decision-making process and to increase the probability of high quality compounds. Drug-likeness is typically used as a rule of thumb intended to identify compounds that fall into the so-called “drug-like” chemical space. A drug-like compound has characteristics that are historically associated with approved drugs, namely good solubility, lipophilicity, membrane permeability and good pharmacophoric features to interact with the binding pocket of the target protein [88]. Several empirical guidelines or rules have been developed: Lipinski’s rule of five (Ro5) [58], Ghose rules [64,65], Veber rules [76] (more commonly known as extended Ro5), and Oprea’s drug-like rules [59,60] (Table 2). Additionally, one can look at similar rules defined for lead-likeness, as well as overall druggable space. However, due to their general nature, it would be interesting to know whether these rules are useful for proteasome inhibitors and, if any, which are the more predictive of activity?

Lipinski’s Ro5 [58], by far the most influential rule, was proposed in 1997, and is related to the prediction of oral bioavailability for compounds that have achieved phase II clinical trials. Lipinski’s rule defines that an orally active compound has no more than one violation of any of the four criteria shown in Table 2 [58,84]. A compound that fails to comply with two or more properties is theoretically more likely to have poor permeation or absorption, leading to poor bioavailability. However multiple exceptions to this continue to occur ever since Ro5 was introduced. It is known that about 6% of drugs orally administered are outside of Lipinski’s space, which means that some therapeutic approaches may include molecules in the “beyond rule of 5 (bRo5)” chemical space. Actually, 21% of new molecules approved by the U.S. Food and Drug Administration (FDA) are bRo5 compounds, in which antineoplastic drugs are also included [90].

First we applied Lipinski’s Ro5 to proteasome inhibitors with marketing authorization, and to three compounds in clinical trials. It is interesting to note that the carfilzomib violates Lipinski’s Ro5 (exceeding weight and HBD), as shown in Table 3, being considered a non-druglike compound, while two other marketed drugs, bortezomib and ixazomib, are well within the Ro5 boundaries. However, oprozomib also fails Lipinski’s drug-like test (also due to high MW), even though it is currently in phase II, which means it obviously shows acceptable pharmaceutical properties (Table 3). Regarding the coverage of drug-like, lead-like and known-drug spaces, bortezomib is exclusively covered by the known-drug space (KDS), carfilzomib is not covered by any of the three spaces and ixazomib is included in all three chemical spaces. Once again, this is evidence of the natural drift from these different chemical spaces defined two decades ago.

**Figure 15:**
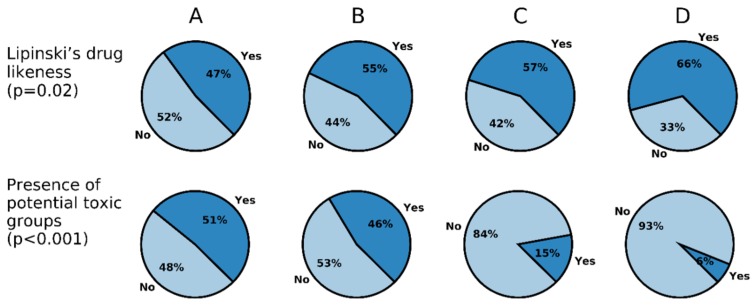
Comparison between the proportion of Lipinski drug-likeness as well as the presence of potential toxic (“mutagenic”) groups across activity classes. The *p*-values were calculated with Fischer’s [93] test adjusting the false discovery rate using the Benjamini-Hochberg procedure [94].

Next we extended the analysis to the dataset of 680 inhibitors of the proteasome CT-L active site. We observed that the percentage of compounds with drug-like properties increases from class A to class D (47 to 66%) (Figure 15). Focusing our analysis on class A compounds, MW is a major cause of non-compliance to Lipinski’s drug-like test, with 79% of compounds having MW > 500. As we mentioned earlier, this rule was highly influential to the drug development community ever since it was published, and there exist different examples of proteasome inhibitor screening projects being driven by its use (Maréchal et al. [91], Lavecchia and Giovanni [92]). However, the increased rate of compounds that are not covered by Ro5 as a function of increased activity can probably be explained by the fact that, even if this filter was applied for early compound selection (therefore covering close to half of all compounds), it ultimately did not influence actual hits found in later stages of screening and optimization. The consistent decrease in the amount of drug-like compounds is likely just a result of the natural expansion from the chemical space used in 1997 to establish the Ro5.

Drug development is biased towards finding new active scaffolds or simply just different compounds from already covered chemical space, which means new actives are more likely to be more dissimilar to the compounds used by Lipinski et al. [58]. These results show that when it comes to proteasome inhibitors, the Ro5 should be applied with some caution, at the risk of stunting the exploration of new regions of chemical space, never forgetting that anticancer drugs are frequently intended for IV rather than oral administration. Overall, the coverage of the various drug-like spaces is quite low for this dataset, with 53% of all compounds obeying Ro5, 22% obeying the extended Ro5, and 0% obeying either Ghose rules or Oprea’s drug-like rules. Additionally, only 11% of all compounds fall inside the Oprea’s lead-like space, and only 51% of compounds are contained in the KDS. This generalized lack of coverage can largely be ascribed to the fact that general drug chemical spaces (as defined by these rules) are fundamentally different to that of proteasome inhibitors, frequently containing very large, peptidomimetic compounds.

Next, we wanted to inspect the data for the presence of potential toxic (“mutagenic”) compounds. This parameter was calculated according to the mutagenic toxicophores prediction defined by Kazius et al. [95] (Figure 15). It shows that the presence of possible toxic groups is predominant in the whole dataset, and as anticipated, it steadily increases from class D to A. This is an expected outcome, since we are in the presence of antineoplastic drugs, which are more likely to have potential toxic/mutagenic groups (e.g., three membered-heterocycles are signaled as toxicophores, but an epoxide is the main functional group of carfilzomib).

### 3.9. Relationship between Functional Groups and Activity Classes

Prompted by the observation that certain groups that are predicted to lead to toxicity are increasingly more prevalent as a function of activity, we analyzed which chemical groups are generally preferred in a proteasome inhibitor project, or alternatively, specifically more/less common in highly active classes. To answer this question we employed a substructural search to characterize the presence of 26 functional groups as defined by Daylight SMARTS, which was done using RDKit.

**Figure 16:**
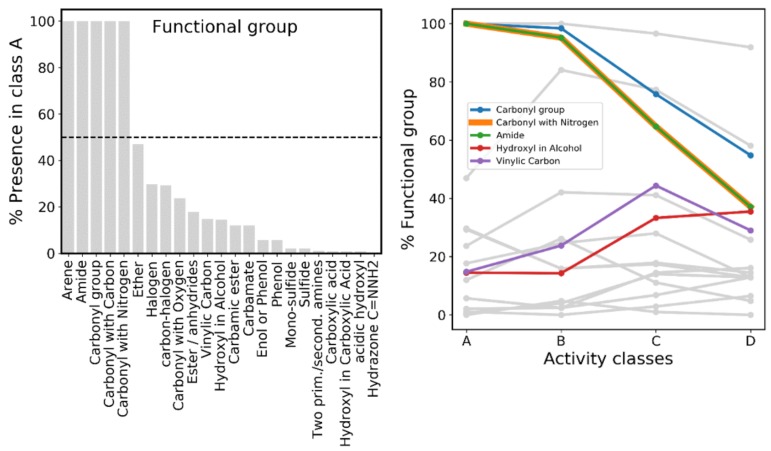
Frequency of common functional groups defined by Daylight SMARTS among proteasome inhibitors. (Left) Frequency (%) of functional groups in class A, sorted by descending order of percentage. Dotted line indicates 50% frequency. (Right) Functional groups highlighted according their (inverse or direct) correlation with activity. The gray lines in the background are the additional functional groups defined in the left plot, and correspond to groups with lower correlation with activity.

Figure 16 (left) shows the frequency of the various functional groups in class A, three of which (amide, carbonyl and carbonyl with nitrogen groups) exist in all compounds within class A, but steadily and markedly decrease with a decreasing activity (Figure 16, right). This is expected, given all three groups are essentially a synonym of, or contained in, peptide (or peptide-like) bonds, which play a central role in allowing interaction within the catalytic sites of the proteasome. Additionally, there are two groups (hydroxyl in alcohol and vinylic carbon) whose frequency shows moderate increase as the activity level decreases (Figure 16, right). 

Given vinyl sulfones are one of the classes of proteasome inhibitors, this inverse correlation with activity indicates that multiple candidates bearing a vinyl carbon were selected for testing with a relatively low success rate, which is a hint that vinyl sulfones are a more challenging class from which to derive new inhibitors. Figure 16 (right) also shows multiple groups with constant low frequency across activity classes (up to a maximum of 20%), among which hydrazone groups, and to a lesser extent, phenol, sulfide and two primary/secondary amine groups, are rare/uncommon across the board. The absence of primary and secondary amines or hydrazone groups is probably a result of the introduction of nitrogen mainly within peptide bonds. As for phenol groups, these have probably a low representation across the board, given the first phenolic ether inhibitors were only introduced in 2013, found by chance though in vitro screening [96]. It is worth noting that these compounds and their derivatives are commonly affected by stability issues only recently addressed in a 2019 study (Yu et al.) [97]. Figure 16 indicates that, on the other hand, ether and arene groups appear to be relatively common across the board as well (ranging between 50% and 80% frequency). For instance, a SAR study in 2007 by Imbach and colleagues [98] revealed several modifications which introduced a benzyl group and lead to high inhibitory activity. This probably influenced the process of candidate design or selection from compound libraries.

### 3.10. Final Remarks on General Guidelines for Drug Design of Proteasome Inhibitors

**Figure 17:**
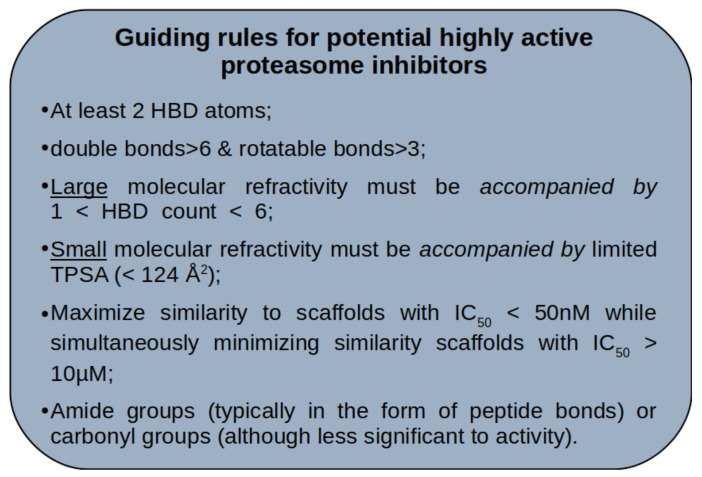
General guidelines for proteasome inhibitors, extracted from the analysis of publicly available inhibition data of the last two decades.

This study allowed the identification of typical chemical patterns associated with potent inhibitory activity against the 20s proteasome, specifically regarding the CT-L catalytic site. We have distilled these into a general guideline for drug design of proteasome inhibitors, summarized in Figure 17. However one should not forget that, as for any rule-of-thumb, these too are general rules. Despite covering a large proportion of the most potent class of compounds, this guideline 1) does not cover all known potent active space and 2) it is not exclusive to potent actives. This set of rules can be used in initial stages of drug design to bias the search towards a region of chemical space which is more likely associated with potent activity.

## 4. Conclusions

After the discovery of bortezomib, the first-in-class proteasome inhibitor, two decades ago, bioactivity data on proteasome inhibitors has continuously grown; however, to date, no attempt has been made to harness this data towards distilling any insight on chemical patterns that drive activity. Prompted by this knowledge gap, the main goal of this study was to identify the key structural and physicochemical determinants of proteasome inhibitors to drive future drug discovery and optimization campaigns. To enable this, we assembled and curated a dataset comprising of 680 compounds divided into four activity classes and with inhibitory activities between 0.08 and 150,000 nM, measured for the CT-L catalytic subunit. Specifically, we calculated and statistically analyzed 21 molecular descriptors that allowed us to map the chemical space occupied by proteasome inhibitors from different classes of potency; we analyzed scaffold similarities, and based on the molecular descriptors, a decision tree was trained.

The determination of the chemical space led to the conclusion that there is a difference in the distribution of the four IC_50_ classes, with the most active compounds (class A) populating a different region of chemical space than the least active compounds (class D). This shows that class D is fundamentally different from the three other classes at a structural and physicochemical level, supporting the existence of a SAR in this dataset. Calculations of the similarity between Murcko scaffolds within each class suggests a high diversity of scaffolds in each class and between the four classes. We found a transition between scaffolds across classes, where class C has a two-peak distribution, which suggests some common scaffolds with classes A/B, as well as with compounds of class D. These data were confirmed through the Venn diagrams of scaffolds which show a decrease in the common scaffolds from class A to D. This activity-related pattern indicates that exploring the modifications of the scaffolds seen in class A, which are simultaneously dissimilar from scaffolds in class D, might be a good strategy for the initial prioritization of screening libraries in the search for new inhibitors.

A decision tree was trained in which we identified two decision rules that allow classifying 63.6% of class A. Both rules efficiently separate class A from the remaining three classes, which is shown by the high purity level (i.e., compounds of class A/all compounds) achieved after the last decision split in each rule (e.g., 86% and 70%, respectively). This observation was followed up by a careful analysis of molecular descriptors which disclosed some trends that drive activity. From our results we saw a decrease in the MW and number of atoms for class A to class D, however surprisingly, only the number of atoms was found as a relevant descriptor in the two main decision rules. As anticipated by the expected interactions in the proteasome CT-L catalytic site binding pocket topology, HBA and HBD were of significant importance, which was translated into a drop-off of the inhibitory potency with the observed decrease of HBA to HBD from class A to D. 

These results confirm the importance of H-Bonds that could stabilize the compounds improving the protein-ligand affinity.

In class A, the HBA and HBD mode in both is 4 (30 being the mode of the number of hydrophobic atoms), allowing this considerable number of atoms to establish the required interactions with the key amino acids (e.g., polar amino acids Thr21 and nonpolar amino acids such as Ala20, Met45, Ala49, Gly47 and Lys52).

Our results show a clear trend with compounds activity for the shape and surface descriptors MR and TPSA, demonstrating that both descriptors slightly increase as proteasome inhibitory activity increases, from class D to A. Giving an overall look of the bond descriptors related with compound flexibility, namely, the number of double and rotatable bounds, our results are peculiar, revealing a structure-activity relationship across classes. What we observe is that both the number of double and rotatable bonds increases as activity increases, from class D to A.

A curious aspect is the existence of only three molecules with triple bonds in the whole database, one in class B and two in class C, giving the idea that their presence would restrict too much the flexibility of the molecules and hamper the interactions in the pocket. In our analyses, there are two descriptors for which we are not able to observe significant changes in the different classes, logP and logS.

We found that the percentage of compounds with drug-like properties (Lipinski’s Ro5), always an important rule to consider in the drug development process, tend to decrease from class D to A, and the presence of toxic groups increases with activity. However, this is not surprising, first due to the tendency to increase the MW during the last years, and also, the toxicity is completely justifiable, since we are dealing with potentially antineoplastic drugs.

Prompted by the observation that certain groups that are predicted to lead to toxicity are increasingly more prevalent as a function of activity, we analyzed which chemical groups are generally preferred. We found that amide, arene and different carbonyl groups are among the most prevalent in those among the most active compounds, and these also decrease in frequency as one transitions from classes A to D. This is a result of the fact that most proteasome inhibitors are peptidomimetic compounds bearing multiple peptide (or peptide-like) bonds.

Finally, evaluating how this dataset is covered by the multiple typically used druggability filters indicates an apparent drift into different locations of chemical space, evident by the overall low coverage by various such filters. The systematic analysis of proteasome inhibitors tested in the last 20 years revealed multiple chemical patterns which can be used to inform future drug discovery projects, effectively helping in biasing the search into known active spaces, as well as into yet unexplored spaces.

## Figures and Tables

**Table 1:** Inhibitors of the human 20S proteasome on market and submitted to clinical trials. The corresponding structures and activities can be found in Figure 2.

Proteasome Inhibitor	Structural Class	Administration Route	Market Authorization	Phase of Ongoing Clinical Trials and Respective Disease(s)
Bortezomib	Boronate	IV, SC	MM, MCL	I, II, III, IV MM, leukemia, myasthenia gravis, systemic lupus erythematosus, rheumatoid arthritis, solid tumors
Carfilzomib	Epoxyketone	IV	MM	I, II, III, IV M, lymphoma, neuroendocrine cancers, solid tumors
Delanzomib	Boronate	IV, PO	–	I (Tested in MM, lymphoma, solid tumors)
Ixazomib	Boronate	IV, PO	MM	I, II, III, IV MM, leukemia, lymphoma
Marizomib	β-Lactone	IV, PO	–	I, II, III MM, glioma, solid tumors, lymphoma, leukemia, lung cancer
Oprozomib	Epoxyketone	IV, PO	–	I, II MM

IV = intravenous, SC = subcutaneous, PO = per os (oral), MM = multiple myeloma, MCL = mantle cell lymphoma.

**Table 2:** Several drug-likeness rules.

	Drug-likeness Rules				Lead-likeness	Marketed Drug Space
Molecular Descriptor	Lipinski [58]	Ghose [64,65]	Veber [76]	Oprea [59,60]	Oprea’s Lead-likeness [60]	Known Drug Space, KDS [89]
MW	≤500	[160; 480]	–	[200; 450]	≤450	≤800
LogP	≤5	[−0.4; 5.6]	–	[−2; 4.5]	[−3.5; 4.5]	≤6.5
HBA	≤10	–	–	[1; 8]	≤8	≤15
HBD	≤5	–	–	≤5	≤5	≤7
Number of atoms	–	[20; 70]	–	–	–	–
MR	–	[40; 130]	–	–	–	–
RotN	–	–	≤10	[1; 9]	≤10	≤17
Rings	–	–	–	≤5	≤4	–
PSA (Å^2^)	–	–	≤140	–	–	≤180
LogD_7.4_	–	–	–	[−4;4]	–	–

MW: molecular weight; logP: octanol–water partition coefficient; HBA: number of hydrogen bond acceptor atoms; HBD: number of hydrogen bond donor atoms; MR: molecular refractivity; RotN: number of rotatable bonds; PSA: polar surface area.

**Table 3:** Molecular descriptors for proteasome inhibitors already with marketing authorization or in clinical trials.

	Compounds with marketing authorization			Compounds in clinical trials		
Molecular Descriptors	Bortezomib	Carfilzomib	Ixazomib	Delanzomib	Marizomib	Oprozomib
MW	384.2	719.9	361.0	413.3	313.8	532.6
LogP (o/w)	0.95	4.08	2.48	1.801	0.774	0.007
HBA	6	8	4	6	3	8
HBD	4	4	4	5	2	3
hydrophobic atoms	17	35	14	19	14	22
MR	11.02	20.18	9.36	11.78	7.85	14.03
Rotatable bonds	11	24	9	11	4	17
Rings	2	4	1	2	3	3
TPSA	124.4	158.5	98.7	131.8	75.6	148.3
Lipinski’s drug-likeness	Yes	No	Yes	Yes	Yes	No
Ro5 violations	0	2*	0	0	0	2*

* Values obtained from HBA/HBD counts as defined by Lipinski et al. [58].

## Supplementary Materials

Supplementary materials can be found at https://www.mdpi.com/1422-0067/20/21/5326/s1.

## References

[1] International Agency for Research on Cancer—World Health Organization (2018). Latest Global Cancer Data: Cancer Burden Rises to 18.1 Million New Cases and 9.6 Million Cancer Deaths in 2018.

[2] Ciechanover A. (2007). Intracellular protein degradation from a vague idea through the lysosome and the ubiquitin-proteasome system and on to human diseases and drug targeting. Ann. N. Y. Acad. Sci..

[3] Ciechanover A. (1998). The ubiquitin–proteasome pathway: On protein death and cell life. EMBO J..

[4] da Fonseca P.C., He J., Morris E.P. (2012). Molecular model of the human 26S proteasome. Mol. Cell.

[5] Borissenko L., Groll M. (2007). 20S proteasome and its inhibitors: Crystallographic knowledge for drug development. Chem. Rev..

[6] Berkers C.R., Leestemaker Y., Schuurman K.G., Ruggeri B., Jones-Bolin S., Williams M., Ovaa H. (2012). Probing the specificity and activity profiles of the proteasome inhibitors bortezomib and delanzomib. Mol. Pharm..

[7] Petrucci M.T., Giraldo P., Corradini P., Teixeira A., Dimopoulos M.A., Blau I.W., Drach J., Angermund R., Allietta N., Broer E. (2013). A prospective, international phase 2 study of bortezomib retreatment in patients with relapsed multiple myeloma. Br. J. Haematol..

[8] Schmitt S.M., Deshmukh R.R., Dou Q.P. (2014). Proteasome inhibitors and lessons learned from their mechanisms of action and resistance in human cancer. Resistance to Proteasome Inhibitors in Cancer.

[9] Kale A.J., Moore B.S. (2012). Molecular mechanisms of acquired proteasome inhibitor resistance. J. Med. Chem..

[10] Ao L., Wu Y., Kim D., Jang E.R., Kim K., Lee D.M., Kim K.B., Lee W. (2012). Development of peptide-based reversing agents for P-glycoprotein-mediated resistance to carfilzomib. Mol. Pharm..

[11] Chiba T., Matsuda A., Ichikawa S. (2015). Structure-activity relationship study of syringolin A as a potential anticancer agent. Bioorg. Med. Chem. Lett..

[12] Hovhannisyan A., Pham T.H., Bouvier D., Qin L., Melikyan G., Reboud-Ravaux M., Bouvier-Durand M. (2013). C1 and N5 derivatives of cerpegin: Synthesis of a new series based on structure-activity relationships to optimize their inhibitory effect on 20S proteasome. Bioorg. Med. Chem. Lett..

[13] Macherla V.R., Mitchell S.S., Manam R.R., Reed K.A., Chao T.H., Nicholson B., Deyanat-Yazdi G., Mai B., Jensen P.R., Fenical W.F. (2005). Structure-activity relationship studies of salinosporamide A (NPI-0052), a novel marine derived proteasome inhibitor. J. Med. Chem..

[14] Zhu Y., Zhu X., Wu G., Ma Y., Li Y., Zhao X., Yuan Y., Yang J., Yu S., Shao F. (2010). Synthesis, in vitro and in vivo biological evaluation, docking studies, and structure-activity relationship (SAR) discussion of dipeptidyl boronic acid proteasome inhibitors composed of B-amino acids. J. Med. Chem..

[15] Wenlock M.C., Barton P. (2013). In *Silico* physicochemical parameter predictions. Mol. Pharm..

[16] da Fonseca P.C., Morris E.P. (2008). Structure of the human 26S proteasome: Subunit radial displacements open the gate into the proteolytic core. J. Biol. Chem..

[17] Kisselev A.F., Garcia-Calvo M., Overkleeft H.S., Peterson E., Pennington M.W., Ploegh H.L., Thornberry N.A., Goldberg A.L. (2003). The caspase-like sites of proteasomes, their substrate specificity, new inhibitors and substrates, and allosteric interactions with the trypsin-like sites. J. Biol. Chem..

[18] Jung T., Grune T. (2012). Structure of the Proteasome.

[19] de Bettignies G., Coux O. (2010). Proteasome inhibitors: Dozens of molecules and still counting. Biochimie.

[20] Jung T., Catalgol B., Grune T. (2009). The proteasomal system. Mol. Asp. Med..

[21] Bedford L., Paine S., Sheppard P.W., Mayer R.J., Roelofs J. (2010). Assembly, structure, and function of the 26S proteasome. Trends Cell Biol..

[22] Lander G.C., Estrin E., Matyskiela M.E., Bashore C., Nogales E., Martin A. (2012). Complete subunit architecture of the proteasome regulatory particle. Nature.

[23] Goldberg A. (2012). Development of proteasome inhibitors as research tools and cancer drugs. J. Cell Biol..

[24] Verbrugge S.E., Scheper R.J., Lems W.F., de Gruijl T.D., Jansen G. (2015). Proteasome inhibitors as experimental therapeutics of autoimmune diseases. Arthritis Res. Ther..

[25] Blackburn C., Gigstad K.M., Hales P., Garcia K., Jones M., Bruzzese F.J., Barrett C., Liu J.X., Soucy T.A., Sappal D.S. (2010). Characterization of a new series of non-covalent proteasome inhibitors with exquisite potency and selectivity for the 20S beta5-subunit. Biochem. J..

[26] Finley D. (2009). Recognition and processing of ubiquitin-protein conjugates by the proteasome. Annu. Rev. Biochem..

[27] Diez-Rivero C.M., Lafuente E.M., Reche P.A. (2010). Computational analysis and modeling of cleavage by the immunoproteasome and the constitutive proteasome. BMC Bioinformatics.

[28] Zhu Y., Zhao X., Zhu X., Wu G., Li Y., Ma Y., Yuan Y., Yang J., Hu Y., Ai L. (2009). Design, synthesis, biological evaluation, and structure-activity relationship (SAR) discussion of dipeptidyl boronate proteasome inhibitors, part I: Comprehensive understanding of the SAR of alpha-amino acid boronates. J. Med. Chem..

[29] Beck P., Dubiella C., Groll M. (2012). Covalent and non-covalent reversible proteasome inhibition. Biol. Chem..

[30] Dou Q.P., Li B. (1999). Proteasome inhibitors as potential novel anticancer agents. Drug Resist. Updat..

[31] European Medicines Agency (2015). EPAR Summary for the Public: Velcade^®^ (Bortezomib).

[32] Raedler L. (2015). Velcade (Bortezomib) Receives 2 New FDA Indications: For Retreatment of Patients with Multiple Myeloma and for First-Line Treatment of Patients with Mantle-Cell Lymphoma. Am. Heal. drug benefits.

[33] Demo S.D., Kirk C.J., Aujay M.A., Buchholz T.J., Dajee M., Ho M.N., Jiang J., Laidig G.J., Lewis E.R., Parlati F. (2007). Antitumor activity of PR-171, a novel irreversible inhibitor of the proteasome. Cancer Res..

[34] Kupperman E., Lee E.C., Cao Y., Bannerman B., Fitzgerald M., Berger A., Yu J., Yang Y., Hales P., Bruzzese F. (2010). Evaluation of the proteasome inhibitor MLN9708 in preclinical models of human cancer. Cancer Res..

[35] Piva R., Ruggeri B., Williams M., Costa G., Tamagno I., Ferrero D., Giai V., Coscia M., Peola S., Massaia M. (2008). CEP-18770: A novel, orally active proteasome inhibitor with a tumor-selective pharmacologic profile competitive with bortezomib. Blood.

[36] Chauhan D., Catley L., Li G., Podar K., Hideshima T., Velankar M., Mitsiades C., Mitsiades N., Yasui H., Letai A. (2005). A novel orally active proteasome inhibitor induces apoptosis in multiple myeloma cells with mechanisms distinct from Bortezomib. Cancer Cell.

[37] Zhou H.J., Aujay M.A., Bennett M.K., Dajee M., Demo S.D., Fang Y., Ho M.N., Jiang J., Kirk C.J., Laidig G.J. (2009). Design and synthesis of an orally bioavailable and selective peptide epoxyketone proteasome inhibitor (PR-047). J. Med. Chem..

[38] Tietsche V., Hungria D.M., De Queiroz E., Isabel R., Maiolino A., José R., Magalhães P., Sobrinho N., Vaz J., Coutinho R. (2019). New proteasome inhibitors in the treatment of multiple myeloma. Hematol. Transfus. Cell Ther..

[39] (2014). EMA-EU/3/14/1295. https://www.ema.europa.eu/en/medicines/human/orphan-designations/eu3141295.

[40] U.S. Food and Drug Administration Orphan Drug Designations and Approvals. https://www.accessdata.fda.gov/scripts/opdlisting/oopd/.

[41] EMBL-EBI ChEMBL24.1 database. 10.6019/CHEMBL.database.24.1.

[42] Chemical Computing Group MOE (2019). Molecular Operating Environment.

[43] Todeschini R., Consonni V. (2009). Molecular Descriptors for Chemoinformatics.

[44] Ioakimidis L., Thoukydidis L., Mirza A., Naeem S., Reynisson J. (2008). Benchmarking the reliability of QikProp. Correlation between experimental and predicted values. QSAR Comb. Sci..

[45] Matuszek A.M., Reynisson J. (2016). Defining known drug space using DFT. Mol. Inform..

[46] Muchmore S.W., Edmunds J.J., Stewart K.D., Hajduk P.J. (2010). Cheminformatic tools for medicinal chemists. J. Med. Chem..

[47] Van der Maaten L., Hinton G. (2008). Visualizing data using t-SNE. J. Mach. Learn. Res..

[48] Pedregosa F., Gael V., Michel V., Thirion B., Grisel O., Blondel M., Prettenhofer P., Weiss R., Duborg V., Vanderplas J. (2011). Scikit-Learn: Machine Learning in Python.

[49] Bemis G.W., Murcko M.A. (1996). The properties of known drugs. 1. Molecular frameworks. J. Med. Chem..

[50] Landrum G. RDKit: Open-source Cheminformatics. https://www.rdkit.org/.

[51] Rogers D.J., Tanimoto T.T. (1960). A computer program for classifying plants. Science.

[52] Rogers D., Hahn M. (2010). Extended-connectivity fingerprints. J. Chem. Inf. Model..

[53] IBM Corp (2017). SPSS Statistics, version 25.0.

[54] Jones E., Oliphant E., Peterson P. (2007). SciPy: Open source scientific tools for python. Comput. Sci. Eng..

[55] Waskom M. Seaborn: Statistical data visualizationn. https://seaborn.pydata.org/.

[56] John D. (2007). Hunter Matplotlib: A 2D graphics environmen. Comput. Sci. Eng..

[57] Kluyver T., Ragan-Kelley B., Pérez F., Granger B., Bussonnier M., Frederic J., Kelley K., Hamrick J., Grout J., Corlay S., Fernando Loizides B.S. (2016). Jupyter notebooks—A publishing format for reproducible computational workflowsIOS Press Ebooks—J. Positioning and Power in Academic Publishing: Players, Agents and Agendas.

[58] Lipinski C.A., Lombardo F., Dominy B.W., Feeney P.J. (1997). Experimental and computational approaches to estimate solubility and permeability in drug discovery and development settings. Adv. Drug Deliv. Rev..

[59] Oprea T.I., Gottfries J., Sherbukhin V., Svensson P., Kühler T.C. (2000). Chemical information management in drug discovery: Optimizing the computational and combinatorial chemistry interfaces. J. Mol. Graph. Model..

[60] Oprea T.I., Davis A.M., Teague S.J., Leeson P.D., Astrazeneca R., Mo D. (2001). Is there a difference between leads and drugs? A historical perspective. J. Chem. Inf. Comput. Sci..

[61] Azad I., Nasibullah M., Khan T., Hassan F., Akhter Y. (2018). Exploring the novel heterocyclic derivatives as lead molecules for design and development of potent anticancer agents. J. Mol. Graph. Model..

[62] Witten I.H., Frank E., Hall M.A., Pal C.J. (2016). Data Mining: Practical Machine Learning Tools and Techniques.

[63] Hann M.M. (2015). Molecular obesity, potency and other addictions in drug discovery. Multifaceted Roles of Crystallography in Modern Drug Discovery.

[64] Ghose A.K., Crippen G.M. (1986). Atomic physicochemical parameters for three-dimensional structure-directed quantitative structure-activity relationships, I. Partition coefficients as a measure of hydrophobicity. J. Comput. Chem..

[65] Ghose A.K., Crippen G.M. (1987). Atomic physicochemical parameters for three-dimensional-structure-directed quantitative structure-activity relationships. 2. Modeling dispersive and hydrophobic interactions. J. Chem. Inf. Model..

[66] Shultz M.D. (2019). Two decades under the Influence of the rule of five and the changing properties of approved oral drugs. J. Med. Chem..

[67] Varma A.K., Patil R., Das S., Stanley A., Yadav L., Sudhakar A. (2010). Optimized hydrophobic interactions and hydrogen bonding at the target-ligand interface leads the pathways of drug-designing. PLoS ONE.

[68] Hayler J., Davis A., Ward S.E. (2015). The Handbook of Medicinal Chemistry: Principles and Practice.

[69] Harshbarger W., Miller C., Diedrich C., Sacchettini J. (2015). Crystal structure of the human 20S proteasome in complex with Carfilzomib. Structure.

[70] Bogyo M., McMaster J.S., Gaczynska M., Tortorella D., Goldberg A.L., Ploegh H. (1997). Covalent modification of the active site threonine of proteasomal β subunits and the Escherichia coli homolog HslV by a new class of inhibitors. Proc. Natl. Acad. Sci. USA..

[71] Baldisserotto A., Marastoni M., Lazzari I., Trapella C., Gavioli R., Tomatis R. (2008). C-terminal constrained phenylalanine as a pharmacophoric unit in peptide-based proteasome inhibitors. Eur. J. Med. Chem..

[72] Faghih M., Daeihamed M., Abdolmajid S. (2018). Design, synthesis and evaluation of substituted Aryl-2-Nitrovinyl derivatives as small molecules proteasome inhibitors. Iran. J. Pharm. Res..

[73] Lorentz H.A. (1880). Ueber die beziehung zwischen der fortpflanzungsgeschwindigkeit des lichtes und der körperdichte. Ann. der Phys. und Chem..

[74] Lorenz L. (1880). Ueber die refractionsconstante. Ann. der Phys. und Chem..

[75] Bahmani A., Saaidpour S., Rostami A. (2017). A simple, robust and efficient computational method for n-Octanol/Water partition coefficients of substituted aromatic drugs. Sci. Rep..

[76] Veber D.F., Johnson S.R., Cheng H.Y., Smith B.R., Ward K.W., Kopple K.D. (2002). Molecular properties that influence the oral bioavailability of drug candidates. J. Med. Chem..

[77] Congreve M., Carr R., Murray C., Jhoti H. (2003). A ‘Rule of Three’ for fragment-based lead discovery?. Drug Discov. Today.

[78] Pilkington L. (2018). Lignans: A chemometric analysis. Molecules.

[79] Lu J.J., Crimin K., Goodwin J.T., Crivori P., Orrenius C., Xing L., Tandler P.J., Vidmar T.J., Amore B.M., Wilson A.G.E. (2004). Influence of molecular flexibility and polar surface area metrics on oral bioavailability in the rat. J. Med. Chem..

[80] Overkleeft H.S., Bos P.R., Hekking B.G., Gordon E.J., Ploegh H.L., Kessler B.M. (2000). Solid phase synthesis of peptide vinyl sulfone and peptide epoxyketone proteasome inhibitors. Tetrahedron Lett..

[81] Kisselev A.F., Van Der Linden W.A., Overkleeft H.S. (2012). Proteasome inhibitors: An expanding army attacking a unique target. Chem. Biol..

[82] Chao T.-H., Manam R., Hagenbuch B., Weiss J., McArthur K., Wahlgren B., Macherla V., Neuteboom S., Enna S., Lloyd G. (2008). Halogen and non-halogen leaving groups enhance the potency of β-lactone proteasome inhibitors: Further investigation into the role of the halogen of NPI-0052 (salinosporamide A). Cancer Res..

[83] Martin E.J., Critchlow R.E. (1999). Beyond mere diversity: Tailoring combinatorial libraries for drug discovery. J. Comb. Chem..

[84] Lipinski C.A., Lombardo F., Dominy B.W., Feeney P.J. (2001). Experimental and computational approaches to estimate solubility and permeability in drug discovery and development settings. Adv. Drug Deliv. Rev..

[85] Arnott J.A., Kumar R., Planey S.L. (2013). Planey lipophilicity indices for drug development. J. Appl. Biopharm. Pharmacokinet..

[86] Lipinski C.A. (2000). Drug-like properties and the causes of poor solubility and poor permeability. J. Pharmacol. Toxicol. Methods.

[87] Keserü G.M., Makara G.M. (2009). The influence of lead discovery strategies on the properties of drug candidates. Nat. Rev. Drug Discov..

[88] Muegge I. (2003). Selection criteria for drug-like compounds. Med. Res. Rev..

[89] Bade R., Chan H.F., Reynisson J. (2010). Characteristics of known drug space. Natural products, their derivatives and synthetic drugs. Eur. J. Med. Chem..

[90] DeGoey D.A., Chen H.-J., Cox P.B., Wendt M.D. (2018). Beyond the rule of 5: Lessons learned from AbbVie’s drugs and compound collection. J. Med. Chem..

[91] Maréchal X., Genin E., Qin L., Sperandio O., Montes M., Basse N., Richy N., Miteva M.A., Vidal J., Villoutreix B.O. (2013). 1, 2, 4-Oxadiazoles identified by virtual screening and their non-covalent inhibition of the human 20S proteasome. Curr. Med. Chem..

[92] Lavecchia A., Giovanni C. (2013). Di virtual screening strategies in drug discovery: A critical review. Curr. Med. Chem..

[93] Fisher R.A. (1922). On the interpretation of χ2 from contingency tables, and the calculation of P. J. R. Stat. Soc..

[94] Benjamini Y., Hochberg Y. (1995). Controlling the false discovery rate: A practical and powerful approach to multiple testing. J. R. Stat. Soc. Ser. B.

[95] Kazius J., Mcguire R., Bursi R. (2005). Derivation and validation of toxicophores for mutagenicity prediction. J. Med. Chem..

[96] Lawrence H.R., Sebti M. (2013). Oxadiazole-isopropylamides as potent and noncovalent proteasome inhibitors. J Med. Chem..

[97] Yu J., Xu L., Hong D., Zhang X., Liu J., Li D., Li J., Zhou Y., Liu T. (2019). Design, synthesis, and biological evaluation of novel phenol ether derivatives as non-covalent proteasome inhibitors. Eur. J. Med. Chem..

[98] Imbach P., Lang M., García-Echeverría C., Guagnano V., Noorani M., Roesel J., Bitsch F., Rihs G., Furet P. (2007). Novel β-lactam derivatives: Potent and selective inhibitors of the chymotrypsin-like activity of the human 20S proteasome. Bioorg. Med. Chem. Lett..

