# Supplementary material for: Chemical Patterns of Proteasome Inhibitors: Lessons Learned from Two Decades of Drug Design
Source: Int J Mol Sci. 2019 Oct 25;20(21):5326. doi: 10.3390/ijms20215326 (PMC6862029; doi:10.3390/ijms20215326)

**Figure S1.** Decision tree built from the proteasome inhibitors dataset, using a decision tree algorithm implemented in scikit-learn.


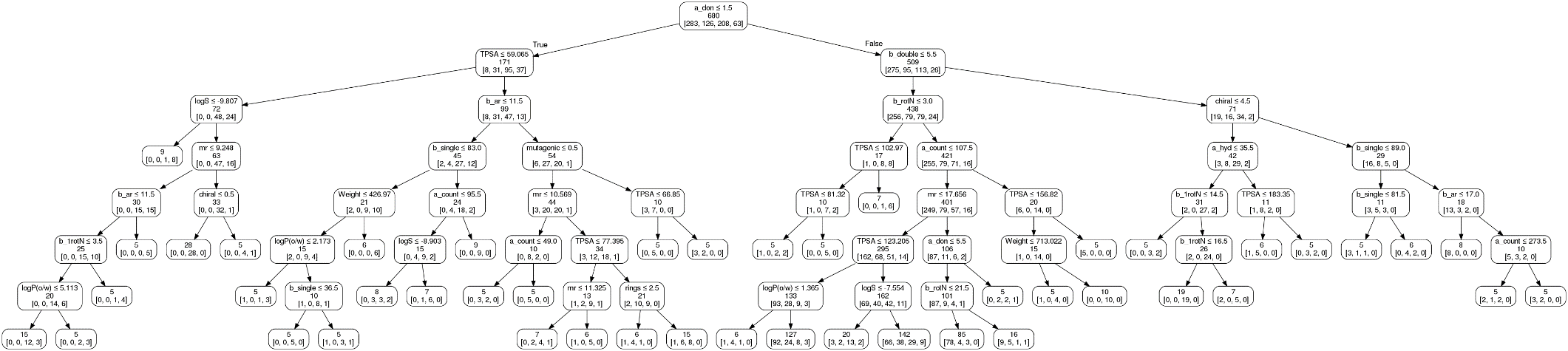

Supplement: Supplementary file 1 [file ijms-20-05326-s001.zip › ijms-625244-SI-to conversion/Figure S1.docx]
